# Health system reform in the context of COVID-19: a policy brief outlining lessons from Ireland’s journey towards the goal of universal healthcare

**DOI:** 10.1186/s41256-025-00407-z

**Published:** 2025-02-26

**Authors:** Sarah Parker, Katharine Schulmann, Carlos Bruen, Sara Burke

**Affiliations:** Centre for Health Policy and Management, Discipline of Public Health and Primary Care, School of Medicine, Trinity College Dublin, Dublin, Ireland

**Keywords:** Universal healthcare, Health system reform, COVID-19, Ireland, Health policy

## Abstract

The COVID-19 pandemic has presented unique challenges and opportunities for health system reform globally. In Ireland, this period coincided with the early stages of the Sláintecare reform plan, a core goal of which is to establish universal healthcare. This policy brief synthesises key research findings from 13 studies carried out under the *Foundations* research programme to harness key learnings from the pandemic response for health system change. The analysis reveals how the COVID-19 crisis accelerated health system reforms in Ireland, breaking from a history of incremental change to implement rapid innovations towards universal healthcare. While a ‘new normal’ has emerged, the challenge remains to integrate these rapid developments into enduring health system improvements under evolving governance and leadership in the COVID-19 context**.** Three significant implications for health systems research and policy are identified: 1) Political consensus is essential for sustained health system reform, particularly during crises; 2) Adaptive health systems that can transform challenges into reform opportunities are crucial; and 3) Co-production in research enhances policy acceptability and implementation by aligning it with real-world complexities. Leveraging these pandemic-driven insights will be key to ensuring that the swift adaptations and lessons learned will transition into lasting elements of Ireland’s health system.

## Background

The COVID-19 pandemic underscored challenges and opportunities for health systems worldwide. In Ireland, the pandemic arrived early in the implementation of health reform efforts that included the planned introduction of universal healthcare. This policy brief synthesises key research findings from 13 studies carried out under the *Foundations* research programme [1], which employed co-production with senior health system decision-makers to harness key reform learnings from the pandemic response (2020–2023). Critical insights into how Ireland’s planned move towards universal healthcare was navigated and impacted during the crisis are presented, offering important research and policy implications for countries undergoing substantial health system transformation.

Unlike most high-income countries, Ireland does not have universal healthcare. This has resulted in a complex, fragmented health system with both public and private financing and provision, where approximately half of the population purchases voluntary health insurance for faster access to certain health services. Eligibility for public healthcare services without charge is largely means tested and while there has been an expansion of the General Practitioner (GP) visit card, there has been a failure to progress a universal entitlement to healthcare. Major organisational reforms in the 1990s culminated in the establishment of the Health Service Executive (HSE), which replaced eight regional Health Boards from 2005 [2]; however, persistently long waitlists and delayed access to care sparked further calls for change. The current reform programme—a ten-year plan called Sláintecare – aims to establish a universal health system based on medical need rather than ability to pay; providing timely, integrated and quality care in the most appropriate setting.

## Research synthesis

Thirteen publications from the *Foundations* research programme were selected for analysis. A synthesis was conducted to capture key findings, using analytic techniques such as interdisciplinary brainstorming, concept mapping and process tracing to track key moments influencing Ireland’s path towards universal healthcare prior to, during and following the COVID-19 crisis (see Table 1).

**Table 1:** Overview of the Foundations research programme studies

Time period	Authors, year	Topic	Study design, method(s)	Aim, context	Findings
Pre COVID-19	Barry et al., 2021	Service reorganisation in health and social care	Qualitative multi-method study including a rapid review, document analysis and elite interviews	Analysed service reorganisation in the Irish health and social care system from 1998 to 2020 to identify lessons for reform implementation. It was commissioned by the Department of Health to inform the design and implementation of the regions under Sláintecare	Effective system-wide change has been hindered by political, organisational and implementation failures, with issues such as poor clarity and commitment to policy and process; weak change management and resourcing; and resistance to altering established practices and cultures within the system
	Johnston et al., 2021	Population-based resource allocation strategies	Qualitative multi-method study including a narrative rapid review and document analysis	Examined the impacts and outcomes of population-based resource allocation (PBRA) across six high-income countries. The work was requested by the Department of Health since PBRA was a recommendation of Sláintecare	Countries showed notable differences in objectives, model descriptions and variable criteria in funding formulae, highlighting tensions in aligning policy goals, model transitions, regionalisation and governance mechanisms. Population-based resource allocation is a key lever to support Ireland’s aim of transitioning to a universal health system
	Thomas et al., 2021	Sláintecare implementation status	Policy analysis	Assessed: 1) how close Sláintecare is to the ideal of Universal Healthcare; 2) the systems thinking approach to transition in Sláintecare; and 3) how Sláintecare has evolved since its publication in May 2017	Progress in expanding entitlements under Sláintecare has been limited, despite some positive developments like integrated care pilots and citizen engagement. However, an emphasis on organisational change risks neglecting essential elements of entitlement expansion
COVID-19 Onset	Burke et al., 2020	*Foundations’* study protocol	Protocol	Outlined the shift in the *Foundations* project from a focus on regionalisation towards seeking to learn from the rapid, and in some instances transformative Irish health system COVID-19 response, to assess and interpret relevance to Sláintecare’s implementation	N/A
	Marron et al., 2021	Acute hospital care utilisation during the pandemic	Quantitative study, including secondary analysis of national data	This collaborative work between the HSE and *Foundations’* research team quantified and characterised changes in Irish acute public hospital utilisation during wave one of COVID-19 to inform healthcare system planning and recovery	The COVID-19 pandemic led to significant reductions in Emergency Department visits, admissions and non-COVID-19 hospital admissions, with the greatest drops during peak restrictions. Post-first wave, while overall hospital utilisation remained low, there was an increase in alcohol-related, self-harm and mental health admissions
	McGlacken-Byrne et al., 2021	Healthcare activity tracking during the pandemic	Quantitative study, including secondary analysis of publicly available data	Explored the impact of COVID-19 on aspects of Irish healthcare during the first nine months of the pandemic and considers the implications for Sláintecare implementation	COVID-19 significantly disrupted healthcare in Ireland, with 3.76 million tests conducted and 2.31 million GP telephone triages by March 2021. Allied healthcare specialties saw a 35.1% drop in patient numbers and hospital waitlists increased by 19.1%, with substantial outpatient backlogs in orthopaedic surgery, ear, nose and throat (ENT) and ophthalmology
Acute COVID-19 Crisis	Fleming et al., 2022	Workforce trends and recovery	Quantitative study including secondary analysis of human resources data	Workforce emerged as a key issue to reform pre COVID-19 and in coping with the pandemic. This study, a collaboration between the Irish Health Service Executive (HSE) and *Foundations’* research team, aimed to uncover workforce trends overtime and assess if they were aligned with Ireland’s aim of universal healthcare, specifically shifting care into primary and community settings	Findings revealed a significant shift in Ireland's healthcare staffing, with acute services consistently prioritised over community care from 2008 to 2021. By 2021, acute settings had over 13,000 more staff than community care, highlighting a misalignment with policy goals to strengthen community-based healthcare
	Burke et al., 2021	Health system resilience in the COVID-19 context	Qualitative study, including documentary analysis	Examined whether and how the Irish government's pandemic response contributed to health system reform and increased resilience including delivering universal healthcare	The COVID-19 pandemic underscored the need for health system resilience. Ireland's Sláintecare reform, aiming for universal healthcare, showed increased policy alignment and budgetary support during the pandemic. This response not only managed the crisis but also advanced health system reforms, offering insights for long-term transformation with international relevance
	Parker et al., 2023	Public health and vulnerable populations during the pandemic	Quantitative study, including secondary analysis of publicly available data	Explored whether and how public health responses to homelessness during COVID-19 hold important insights for the development of more effective health policy, identifying relevant lessons for health reform	Ireland's integrated COVID-19 response not only protected (most) homeless populations from infection but also reduced the number of homelessness presentations, underscoring the need to integrate housing and health policies post-pandemic
	Mac Conghail et al., 2024	Universal access to hospitals during the pandemic	Qualitative multi-method study, including document analysis and key informant interviews	Examined the policy process underlying the historic Safety Net Agreement (SNA) that provided public access to private hospitals, deriving insights for the implementation of universal hospital care	Ireland's COVID-19 response included waiving hospital charges and implementing universal access to hospital care through the SNA, but faced challenges with private hospital consultants, highlighting strained public–private relationships and the importance of robust governance and trust-building for effective public–private healthcare collaborations
	Parker et al., 2023	Access to integrated care during the pandemic	Qualitative study, including semi-structured interviews	Examined how and to what extent COVID-19 highlighted opportunities for reform that enabled better access to universal, integrated care in Ireland, with a view to informing universal health system implementation	The flexible system conditions generated by the pandemic response fostered new integrated care trajectories through establishing a common goal; sharing information; enabling innovations; and prioritising trust, relationship-building and non-hierarchical collaboration. However, an apparent reversion to traditional priorities and procedures will undermine the system’s capacity to sustain these changes
COVID-19 Context	Schulmann et al., 2024	The role of governance in shaping health system reform	Qualitative multi-method study, including document analysis and key informant interviews	Explored the governance of the design of new health regions in Ireland, 2018–2023, in order to better understand how aspects of governance impact on the reform, from policy design through to implementation	Key governance issues identified include deficiencies in accountability, poor transparency, unclear roles and responsibilities and inadequate resourcing. These factors created mistrust among institutional actors and emphasise the importance of strengthening governance arrangements for effective health system reform
	Burke et al., 2024	Learning from economic and public health ‘shocks’ for health system resilience	Qualitative study, including document analysis	Examined Ireland’s experiences of the economic (2008–2014) and COVID-19 pandemic (2020–2023) shocks and their impact on the current and future resilience of the Irish health system	Despite slow progress, Ireland’s COVID-19 response aligned with Sláintecare, highlighting the need for a comprehensive, adaptive approach to build enduring health system resilience and address past policy failures

*Foundations* research covering the pre COVID-19 period from 1998 to early 2020 outlines a slow, shaky trajectory towards universal healthcare [2]. Published in 2017, Sláintecare signalled political consensus on the goal of universal healthcare, but wavering support affected its rollout pre-2020, particularly in terms of promised entitlement expansions and service delivery reorganisation [3]. Policy intent to develop population-based resource allocation showed potential [4], however efforts to reduce prescription charges and expand free GP care were inconsistent during this time. Despite sustained if somewhat muted political endorsement and funding allocations in government budgets from 2017 to 2019, the 2018 implementation plan lacked clarity and sufficient financial backing [3].

The arrival of COVID-19 in March 2020 further disrupted early reform efforts, at least initially, prompting a rapid shift in the health system’s focus [1]. During the first phase of COVID-19, Ireland’s health system priorities shifted to public health and taking non-acute care out of hospitals to free up capacity for COVID-19 and acute/specialist outpatient care. However, the response was impacted by legacy workforce allocation issues, in part underpinned by workforce shortages prior to the pandemic as well as decades of acute care prioritisation over community settings. Although overall staffing increased by 8.9% during the pandemic, there were 13,645 more publicly-funded workers in acute settings compared to the community by August 2021 [5]. This imbalance meant that primary care and community-based services faced workforce and redeployment challenges, indicating a lack of commitment to recruit the necessary staff-mix to achieve a universal health system and highlighting the need to revisit workforce strategies.

This initial period was the pressure point that revealed Ireland’s health system weaknesses by intensifying pre-existing problems such as waitlists for acute and specialist outpatient care and barriers to healthcare access. Public hospital waiting lists increased by 19.1%, from 729,937 in January 2019 to 869,676 in January 2021; 629,919 patients awaited a first outpatient appointment, with 27.1% waiting over 18 months; and 3.76 million COVID-19 tests were conducted with GPs delivering 2.31 million telephone triages by March 2021, contributing to professional burnout [6]. Additionally, new challenges emerged including the public health implications of delayed and lost care during the first wave in 2020 (e.g. 81,712 fewer Emergency Department [ED] presentations and 210,357 fewer non-COVID-19 hospital admissions) as well as increases in emergency alcohol-related, self-harm and mental health admissions [7]. Together, this highlighted the urgent need for a universal, resilient health system capable of addressing new, evolving population health needs as well as future pandemics [8].

At the same time, other areas of Ireland’s response demonstrated system adaptions with potential to drive universal health reform forward. This included a universal community-based approach to COVID-19 care and temporary policy measures to mitigate Ireland’s complex public/private divide to ensure equitable care access [9]; core aims of the Sláintecare reform programme. Specifically, COVID-19 was classified as a notifiable disease, exempting all patients from public hospital charges for treatment of symptoms; the government also negotiated the public take-over of private hospitals for the first three months of COVID-19 and offered private-only medical consultant contracts to work in the public system [9]. However, this arrangement faced criticism for its cost and underutilising capacity, leading to its termination after three months.

Ireland’s pandemic response also led to significant health system changes that enabled agile and rapid solutions through increased funding, relaxed procurement processes and fast-tracked digital health responses [10]. These more flexible conditions fostered trust and collaboration within and across the health system, resulting in innovations to improve access to integrated care such as the use of telemedicine, enhanced communication channels and rapid adaptation of care models [10]. Successes were also observed in responses targeting certain populations considered ‘vulnerable’, including those accessing homelessness services. Measures including eviction bans and the relocation of service users into single occupancy housing helped to reduce infection rates, COVID-19 related deaths and homelessness presentations [11]. Nonetheless, the sustainability/continuation of the system changes implemented during the crisis remains uncertain [10, 11]. Moreover, clarity regarding entitlements and poor healthcare access continue to be major concerns despite the recent abolition of in-patient hospital charges, expansion of eligibility for free GP services and a new public-only contract for medical consultants [12].

Finally, developments since 2022 suggest a gradual return of policy attention to, and capacity for, the decentralisation of health care delivery [13]. However, governance challenges such as accountability deficiencies, poor transparency, unclear roles and responsibilities, inadequate implementation capacity and competing policy visions, have prompted reflection on the role and definition of universalism within the new regional framework; namely, whether it continues to be a central goal of health reform and the level of commitment to universalism in the COVID-19 context [13].

The narrative account demonstrates how crises can be powerful catalysts for change. The pandemic jolted Ireland’s health system from its path of incremental reform, forcing rapid adaption and innovation. This echoes a punctuated equilibrium approach to understanding policy-making processes, where long periods of stability are interrupted by brief, intense periods of significant change. The pandemic represents a punctuation point, propelling the system towards necessary reforms and innovations that might otherwise have taken decades to achieve. While a ‘new normal’ for Ireland’s trajectory towards universal healthcare emerged, past practices and political stances regarding its desirability/viability continue to shape new governance, leadership and service-delivery arrangements in 2025. The question now is whether Ireland will solidify the lessons learned during the pandemic to ensure foundational elements of universal healthcare become permanent, enduring fixtures of the health system, rather than fleeting crisis measures.

## Implications for health systems research and policy

- *Political consensus is crucial for sustaining health system reform agendas both during and outside of crisis periods -* In the face of health crises and political changes, an agreed, cross-party political commitment to health reform lays a critical foundation on which a universal health system can be built over time. A unified vision on health reform goals provides continuity and guides policy evolution, keeping long-term objectives in focus even as strategies and priorities shift to meet immediate or future challenges.
- *An adaptive health system can leverage crises into reform opportunities*—Amid crises, an adaptive health system can do more than survive; it can reorient challenges into potentially transformative opportunities. Building on this, health systems should prioritise readiness/agility by incorporating robust planning, flexible resource allocation, an integrated workforce and the capacity for rapid innovation.
- *Optimising co-production in research on health system reform requires strategic management*—Engaging stakeholders and knowledge users sensitises research to the real-world complexities of health system reform, enhancing the acceptability and uptake of findings. However, co-production must be carefully managed to maximise its benefits while mitigating its limitations, particularly in terms of reaching consensus on differing research priorities and interpretations.

## Conclusions

The findings from the *Foundations* research programme emphasise the urgency of leveraging the pandemic’s challenges as opportunities for systemic health reform. Ireland's experience offers valuable lessons for building more resilient, inclusive and innovative health systems worldwide. Grounded in empirical research and aligned with practical policy implications, this policy brief lays the foundations for informed policy development and future research, contributing to the global dialogue on health system reform in the pandemic recovery era.

## Data Availability

The datasets generated and/or analysed during the current study are not publicly available due to the sensitive nature if the research, but are available from the corresponding author on reasonable request.

## References

[1] Burke S, Thomas S, Stach M, Kavanagh P, Magahy M, Johnston B, Barry S. Health system foundations for Sláintecare implementation in 2020 and beyond? Co-producing a sláintecare living implementation framework with evaluation: learning from the Irish health system’s response to COVID-19 mixed-methods study protocol. HRB Open Res. 2020. 10.12688/hrbopenres.13150.1.2. 33728398 10.12688/hrbopenres.13150.1 PMC7934093

[2] Barry S, Stach M, Thomas S, Burke S. Understanding service reorganisation in the Irish health and social care system from 1998 to 2020: lessons for reform and transformation. HRB Open Res. 2021. 10.12688/hrbopenres.13342.1.34909578

[3] Thomas S, Johnston B, Barry S, Siersbaek R, Burke S. Sláintecare implementation status in 2020: Limited progress with entitlement expansion. Health Policy. 2021;125(3):277–83. 10.1016/j.healthpol.2021.01.009. 33531170 10.1016/j.healthpol.2021.01.009 PMC9757858

[4] Johnston BM, Burke S, Kavanagh PM, O’Sullivan C, Thomas S, Parker S. Moving beyond formulae: a review of international population-based resource allocation policy and implications for Ireland in an era of healthcare reform. HRB Open Res. 2021;4:121. 10.12688/hrbopenres.13453.1.

[5] Fleming P, Thomas S, Williams D, Kennedy J, Burke S. Implications for health system reform, workforce recovery and rebuilding in the context of the great recession and COVID-19: a case study of workforce trends in Ireland 2008–2021. Hum Res Health. 2022;20(1):48. 10.1186/s12960-022-00747-8. 10.1186/s12960-022-00747-8 PMC9134726 35619111

[6] McGlacken-Byrne D, Parker S, Burke S. Tracking aspects of healthcare activity during the first nine months of COVID-19 in Ireland: a secondary analysis of publicly available data. HRB Open Res. 2023;4:98. 10.12688/hrbopenres.13372.2. 10.12688/hrbopenres.13372.3 PMC11439124 39347503

[7] Marron L, Burke S, Kavanagh P. Changes in the utilisation of acute hospital care in Ireland during the first wave of the COVID-19 pandemic in 2020. HRB Open Res. 2022. 10.12688/hrbopenres.13307.3. 36204710 10.12688/hrbopenres.13307.1 PMC9513415

[8] Burke S, Parker S, O’Donoghue C, Thomas S, Barry S, Fleming P. Learning from the economic shock–health system resilience in Ireland. In: Thomas Steve, Fleming Padraic, editors. Handbook of health system resilience. Edward Elgar Publishing; 2024. p. 230–47. 10.4337/9781803925936.00025.

[9] Conghail LM, Parker S, Burke S. Examining universal access to acute hospital care in Ireland during the first three months of COVID-19: lessons from the policy process. HRB Open Res. 2024;7:4. 10.12688/hrbopenres.13848.1. 39927194 10.12688/hrbopenres.13848.1 PMC11803191

[10] Parker S, Mac Conghail L, Siersbaek R, Burke S. How to not revert to type: complexity-informed learnings from the pandemic response for health system reform and universal access to integrated care. Front Public Health. 2023. 10.3389/fpubh.2023.1088728. 36908402 10.3389/fpubh.2023.1088728 PMC9996344

[11] Parker S, Siersbaek R, Mac Conghail L, Burke S. Public health responses to homelessness during COVID-19 in Ireland: implications for health reform. Int J Homelessness. 2023;3(3):36–52. 10.5206/ijoh.2023.3.14967.

[12] Burke S, Parker S, Fleming P, Barry S, Thomas S. Building health system resilience through policy development in response to COVID-19 in Ireland: from shock to reform. Lancet Reg Health Eur. 2021. 10.1016/j.lanepe.2021.100223. 34642676 10.1016/j.lanepe.2021.100223 PMC8495249

[13] Schulmann K, Bruen C, Parker S, Siersbaek R, Mac Conghail L, Burke S. The role of governance in shaping health system reform: a case study of the design and implementation of new health regions in Ireland, 2018–2023. BMC Health Serv Res. 2024;24(1):578. 10.1186/s12913-024-11048. 38702678 10.1186/s12913-024-11048-2 PMC11069256

